# Potential value of the current mental health monitoring of children in state care in England

**DOI:** 10.1192/bjo.2018.70

**Published:** 2018-11-14

**Authors:** Christine Cocker, Helen Minnis, Helen Sweeting

**Affiliations:** Senior Lecturer in Social Work, School of Social Work, University of East Anglia, UK; Professor of Child and Adolescent Psychiatry, Institute of Health and Wellbeing, University of Glasgow, UK; Reader, MRC/CSO Social & Public Health Sciences Unit, University of Glasgow, UK.

**Keywords:** Looked-after children, mental health, screening, Strengths and Difficulties Questionnaire

## Abstract

**Background:**

Routine screening to identify mental health problems in English looked-after children has been conducted since 2009 using the Strengths and Difficulties Questionnaire (SDQ).

**Aims:**

To investigate the degree to which data collection achieves screening aims (identifying scale of problem, having an impact on mental health) and the potential analytic value of the data set.

**Method:**

Department for Education data (2009–2017) were used to examine: aggregate, population-level trends in SDQ scores in 4/5- to 16/17-year-olds; representativeness of the SDQ sample; attrition in this sample.

**Results:**

Mean SDQ scores (around 50% ‘abnormal’ or ‘borderline’) were stable over 9 years. Levels of missing data were high (25–30%), as was attrition (28% retained for 4 years). Cross-sectional SDQ samples were not representative and longitudinal samples were biased.

**Conclusions:**

Mental health screening appears justified and the data set has research potential, but the English screening programme falls short because of missing data and inadequate referral routes for those with difficulties.

**Declaration of interest:**

None.

The mental health of children in state care is of great concern. Because of this, in 2009 the Department for Education introduced compulsory mental health data collection for these children in England by using the Strengths and Difficulties Questionnaire (SDQ). This article examines the degree to which the current mass data collection achieves screening aims and the potential analytic value of the resulting data set.

## The SDQ

The SDQ is an internationally validated questionnaire^1^^,^^2^ comprising 25 items, which are broken down into five scales: emotional symptoms, conduct problems, hyperactivity, friendship/peer problems and pro-social behaviour. A general difficulties score is created by adding up the scores from the first four scales. The cut-offs for this score were originally chosen ‘so that roughly 80% of children in the community are normal, 10% are borderline, and 10% are abnormal’.^3^ There are (almost identical) versions for completion by: parents/carers/teachers of 4- to 16-year-olds, parents/carers/teachers of 3- to 4-year-olds and 11- to 16-year-olds themselves. In addition to high specificity (80%) and sensitivity (85%),^4^ the main benefits of the SDQ are that it is free, quick and straightforward to use.^5^ However, cross-informant agreement tends to be lower for internalising than for (more observable) externalising behaviours,^6^ and emotional symptoms are best identified by self-reports.^7^ The SDQ is one of the most used and recognised child and adolescent screening tools.^5^ In the UK, it has been successfully used to screen for child psychiatric disorders in both community^8^ and looked-after children samples, with the study of looked-after children concluding that ‘screening with the SDQ (carer and teacher versions) could improve the detection and treatment of behavioural, emotional, and concentration problems among looked-after children’^4^ (p. 30).

## Data collection for looked-after children in England

In England, it is compulsory to collect mental health data (using the carer-report SDQ) from all children aged 4/5–16/17 who have been in state care for 1 year or longer.^9^ The mental health of these children is known to be poor^10^ and routine SDQ data is seen by the Department for Education as both a way of identifying ‘the scale of the problem’ and, at an individual level, of highlighting ‘the likelihood that the child either has, or could develop significant mental health problems’^11^ (p. 125). The Department for Education recommends it ‘is used to help decision-making about links with Child and Adolescent Mental Health Services (CAMHS)’; suggests that ‘In the longer term, data from SDQ returns will give an indication on how effective the service provision provided is in meeting the needs of looked after children’ (p. 125) and notes that over time ‘records can show a child's progress – whether difficulties identified remain or, if appropriate interventions have been put in place, whether they have eased’^11^ (p. 128). The Department for Education's aim thus seems to be to use the SDQ in multiple ways: as an indicator of those children and young people (CYP) who are at greater risk than the general population of developing mental health problems; as an outcome measure to monitor the impact of services; and to track CYP who are in the care of the state over time. Since routinely collected demographic, health and placement variables are included with the SDQ in the data set (English SSDA903) it is also a potential source of rich longitudinal data for researchers. SDQs are not completed at entry into care, which rules out before–after analyses; however, it should be possible to use the data set to track demographic, health and/or placement correlates of changes in scores over time. As far as we are aware, this is the first time analyses such as these have been done.

## Aims

This article examines:

- the degree to which the current programme has achieved the intention of providing screening to identify the scale of the problem and whether it has had impact on the mental health of looked-after children in England;
- the potential value of analysing the data set created by that programme.

## Method

### Examining population trends

A common first step in evaluating screening programmes is to examine population trends (e.g. time trends of breast cancer mortality to assess the impact of mammographic screening^12^). The SDQ is an indicator of the prevalence of disorders.^9^ Therefore, one way to evaluate screening of looked-after CYP is to examine aggregate, population-level trends in the SDQ scores over time. In this case, the screening ‘intervention’ is also the outcome measure and, if screening had a positive impact on practice (e.g. leading to effective referral and treatment), we might expect this to be reflected in reduced population SDQ scores over time. Publicly available aggregated data (for example^13^) allowed us to examine population trends in the annual SDQ returns for the 9 years (2009–2017) for which data were available. These include the number of valid SDQ returns (overall and for individual local authorities); percent of those eligible with a return; the mean total difficulties score (range 0–40); and percentages with ‘normal’ (0–13), ‘borderline’ (14–16) and ‘abnormal’ scores (17–40).

### Examining representativeness

To accurately identify the scale of the problem, the SDQ data set would either need to have 100% coverage (the aim of the SSDA903 data collection) or cover a representative sample. To investigate representativeness, we conducted analyses based on the English SSDA903 data set provided, on request, by the Department for Education, which included SDQ data from 2009 to 2012. This comprised individualised data (including demographic and placement-related variables as well as the SDQ) collected annually from every English local authority relating to all CYP who had been looked after continuously for a year or longer at 31 March of the year in question. We compared selected key characteristics of children aged 4–17 with and without an SDQ to determine the representativeness of those with an SDQ data return.

### Examining attrition

For meaningful longitudinal analyses (based on a data set linking individual children over 2 or more years), a representative sample needs to retain sufficient numbers over time and the characteristics of those retained compared with those lost to follow-up should be known. We selected children with a 2009 SDQ return and examined the proportion retained and whether those retained over time differed from those lost. We did this by comparing the 2009 (baseline) characteristics of children with and without longitudinal data over 2, 3 and 4 consecutive years.

### Ethical approval

The study was approved by the University of Glasgow Medical Faculty Ethics Committee (2011/FM06009). Data were provided by the Department for Education Data Services Group in 2011 and 2014.

## Results

### Population trends

**Fig. 1:**
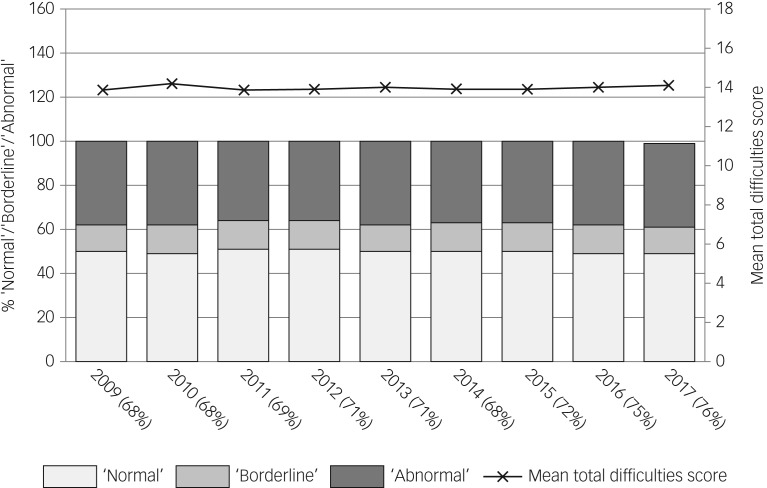
Summary of Department for Education SDQ aggregated data 2009–2017. Percentage with ‘normal’, ‘borderline’ and ‘abnormal’ scores (left-hand axis); mean total difficulties score (right-hand axis); *x*-axis shows percent SDQ returns from those eligible in each year.

Figure 1 (based on Table 1) shows SDQ completion rates and scores from 2009 to 2017. Since the introduction of compulsory data collection, the mean SDQ score has remained consistently close to 14, with around half all children screened falling within the abnormal or borderline score categories. Levels of missing data were around 30% each year from 2009 to 2015 and 25% in 2016–2017. Table 1 shows the range of local authority data-return rates from 2009 to 2016 (2017 data by local authority not available). In 6 of the 8 years, a small number of local authorities submitted no returns; however, over this period the percentage of local authorities submitting returns for 66% or fewer eligible children decreased from 34.4% in 2009 to 21% in 2016.

**Table 1:** Summary of Department for Education SDQ aggregated data over 7 years (2009–2017)

	2009^a^	2010^a^	2011^a^	2012^b^	2013^b^	2014^b^	2015^b^	2016^b^	2017^b^
Number of valid SDQ returns	22 700	22 810	23 870	23 480	24 080	23 650	26 020	27 610	28 810
Percentage of those eligible with SDQ returns	68%	68%	69%	71%	71%	68%	72%	75%	76%
Percentage of local authorities submitting returns for:^c^									
0% eligible children	3.2%	0.0%	0.0%	3.3%	3.3%	2.0%	2.0%	2.0%	^d^
1–33% eligible children	6.5%	3.2%	5.2%	2.6%	2.0%	7.9%	4.6%	2.6%	^d^
34–66% eligible children	24.7%	27.9%	29.2%	23.7%	28.3%	25.7%	16.4%	16.4%	^d^
67–99% eligible children	50.6%	61.7%	59.1%	65.7%	59.9%	56.6%	70.4%	75.7%	^d^
100% eligible children	2.6%	2.6%	1.9%	2.6%	5.3%	5.9%	4.6%	2.6%	^d^
>100% eligible children^e^	9.1%	0.0%	0.0%	0.0%	0.0%	0.0%	0.0%	0.0%	^d^
Described as ‘not applicable’	3.2%	4.5%	4.5%	2.0%	1.3%	2.0%	2.0%	0.7%	^d^
Mean SDQ difficulties score^f^	13.9	14.2	13.9	13.9	14.0	13.9	13.9	14.0	14.1
Percentage of children with:^f^									
‘normal’ score^f^	50%	49%	51%	51%	50%	50%	50%	49%	49%
‘borderline’ score^f^	12%	13%	13%	13%	12%	13%	13%	13%	12%
‘abnormal’ score^f^	38%	38%	37%	37%	38%	37%	37%	38%	38%

SDQ, Strengths and Difficulties Questionnaire.

a. 2009–11 sample stated as aged 4–16.

b. 2012–17 sample stated as aged 5–16.

c. Based on a total of 154 local authorities in 2009–2011 and 152 local authorities in 2012–2017 (from 2012 Cheshire and Bedfordshire ceased to exist as separate authorities).

d. 2017 data by local authority not available.

e. In 2009 there was an anomaly with the data returns and a small number of local authorities returned more than 100% of data.

f. SDQ range 0–40; categorised as 0–13 = normal, 14–16 = borderline and 17–40 = abnormal.^3^

### Representativeness

Table 2 compares the characteristics of the CYP about whom data returns were and were not made in 2009 (results similar for subsequent years). It shows that those with an SDQ were significantly (all *P* < 0.000) more likely to be white (59% compared with 55%), in the middle of the age range (67% of 11- to 15-year-olds compared with only 39% of 16- to 17-year-olds), have no disability (59% compared with 45%) and to be fostered (64% compared with around 50% living in adoption, temporary or residential accommodation; 39% with parents; 17% living independently).

**Table 2:** Characteristics of those with and without an SDQ data return in 2009 (4–17 year olds)

	All (*n*)	SDQ data return	No SDQ return
Overall^a^	38 887	22 681	16 206
		58.3%	41.7%
Gender			
Male	22 231	57.8%	42.2%
Female	16 656	59.0%	41.0%
χ^2^ (significance)		5.2 (0.023)	
Ethnicity			
White	30 447	59.3%	40.7%
Black and minority ethnic	8 440	54.8%	45.2%
χ^2^ (significance)		56.3 (0.000)	
Age			
4	1267	46.5%	53.5%
5–10	9898	64.1%	35.9%
11–15	17 252	67.4%	32.6%
16–17	10 470	39.4%	60.6%
χ^2^ (significance)		2332.5 (0.000)	
Disability^b^			
None	37 182	58.9%	41.1%
Any	1705	45.3%	54.7%
χ^2^ (significance)		123.8 (0.000)	
Placement^c^			
Adoption and temporary placement	1244	48.5%	51.5%
Foster	27 871	64.0%	36.0%
Residential	5519	52.3%	47.7%
Parents	2859	38.7%	61.3%
Independent	1313	17.4%	82.6%
χ^2^ (significance)		1862.7 (0.000)	

SDQ, Strengths and Difficulties Questionnaire.

a. The data set we received included 22 681 children (58.3% of total 38 887), aged 4–17 with an SDQ return. This included 21 669 (64.5% of 33 606) children aged 4–16 and 22 092 (58.7% of 37 620) aged 5–17 with a return. We assume the 22 700 valid SDQ returns in the Department for Education 2009 summary figures shown in Table 1 is the result of rounding, but the 58.3% return rate in the data set does not tally with the 68% figure provided in the summary figures. However, 22 681 is 67.5% of the number of 4- to 17-year-olds in the data set. It is therefore possible that the 2009 Department for Education return rate is based on a numerator of SDQ returns from 4- to 17-year-olds and a denominator of total 5- to 17-year-olds. Government publications themselves are inconsistent in this respect, with one noting both that ‘This indicator [was] … completed for just 65 per cent of the eligible cohort’ and, later in the same document, that ‘SDQ scores were only submitted for 59% of eligible children’.^14^

b. Disability was defined as the reason for entry into care rather than whether or not the child has a disability. It is therefore likely to only identify children who have profound needs.

c. There were 81 cases of missing data on ‘placement’, these were excluded.

### Attrition

Table 3 compares the 2009 (baseline) characteristics of children with and without longitudinal data over 2, 3 and 4 consecutive years. Of those with an SDQ return in 2009, 64% were retained for 2 years (2009–2010), whereas only 28% were retained for 4 years (2009–2012). Those retained in the longitudinal data set were similar to those lost in respect to gender and ethnicity, but they were significantly (all *P* < 0.000) less likely to have had a disability, and were more likely to have been in foster care and to have had an abnormal score in 2009. For example, those retained from 2009 to 2012 included 28% of those with no disability compared with 17% with a disability, and 30% whose 2009 SDQ scores were abnormal compared with 26% whose scores were normal. Children with SDQ returns over consecutive years are therefore not representative of those with an SDQ in any 1 year.

**Table 3:** Characteristics of those with and without SDQ data for 2, 3 and 4 consecutive years

	*N*	2 years SDQ (2009–2010)		3 years SDQ (2009–2011)		4 years SDQ (2009–2012)	
		Yes	No	Yes	No	Yes	No
Overall	22 681	14 515	8166	9628	13 053	6354	16 327
		64.0%	36.0%	42.4%	57.6%	28.0%	72.0%
Gender							
Male	12 857	64.3%	35.7%	43.1%	56.9%	28.4%	71.6%
Female	9824	63.5%	36.5%	41.7%	58.3%	27.5%	72.5%
χ^2^ (significance)		1.6 (0.209)		4.4 (0.036)		1.9 (0.168)	
Ethnicity							
White	18 059	64.5%	35.5%	43.0%	57.0%	28.2%	71.8%
Black and minority ethnic	4622	62.0%	38.0%	40.2%	59.8%	27.3%	72.7%
χ^2^ (significance)		9.7 (0.002)		11.6 (0.001)		1.6 (0.201)	
Age in 2009							
4	589	54.2%	45.8%	32.9%	67.1%	21.9%	78.1%
5–10	6344	70.6%	29.4%	53.4%	46.6%	42.4%	57.6%
11–15	11 623	76.4%	23.6%	52.0%	48.0%	30.4%	69.6%
16–17	4125	20.2%	79.8%	0.0%	100.0%	0.0%	100.0%
χ^2^ (significance)		4363.6 (0.000)		3811.4 (0.000)		2300.6 (0.000)	
Disability in 2009^a^							
None	21 908	64.4%	35.6%	42.9%	57.1%	28.4%	71.6%
Any	773	53.3%	46.7%	30.3%	69.7%	17.2%	82.8%
χ^2^ (significance)		39.7 (0.000)		48.6 (0.000)		46.4 (0.000)	
Placement in 2009^b^							
Adoption and temporary placement	603	18.4%	81.6%	7.0%	93.0%	3.5%	96.5%
Foster	17 840	69.0%	31.0%	47.5%	52.5%	32.3%	67.7%
Residential	2888	52.9%	47.1%	28.4%	71.6%	14.1%	85.9%
Parents	1106	49.0%	51.0%	26.0%	74.0%	15.2%	84.8%
Independent	228	7.0%	93.0%	0.0%	100.0%	0.0%	100.0%
χ^2^ (significance)		1321.8 (0.000)		1019.5 (0.000)		796.7 (0.000)	
SDQ score in 2009							
Normal range	11 344	61.7%	38.3%	40.6%	59.4%	26.2%	73.8%
Borderline range	2793	63.8%	36.2%	41.9%	58.1%	27.9%	72.1%
Abnormal range	8544	67.1%	32.9%	45.1%	54.9%	30.5%	69.5%
χ^2^ (significance)		62.0 (0.000)		40.2 (0.000)		43.9 (0.000)	
SDQ difficulties score in 2009							
Mean score	13.9	14.3	13.2	14.4	13.5	14.6	13.6
*F* (significance)		87.0 (0.000)		63.9 (0.000)		61.8 (0.000)	

SDQ, Strengths and Difficulties Questionnaire.

a. Disability was defined as the reason for entry into care rather than whether or not the child has a disability. It is therefore likely to only identify children who have profound needs.

b. There were 16 cases with missing data on ‘placement’, these were excluded.

## Discussion

Examination of the English SSDA903 data set shows no change in levels of mental health problems in looked-after children since routine screening was introduced in 2009. We found significant levels of missing data and poorly representative cross-sectional and longitudinal samples.

### Whether to screen for mental disorders in looked-after children

Given these findings, a first reaction might be to ask whether SDQ screening of looked-after children is justified. Screening programmes are ‘designed to detect early signs of disease in the population and then to provide a reliable method of referral for diagnostic testing and further treatment’.^15^ The following ten ‘influential principles’^16^ (first published in 1968 and described as ‘a public health classic’^17^) have been widely used to consider whether to screen populations for noninfectious diseases: the condition should be an important health problem, there should be an accepted treatment, facilities for diagnosis and treatment should be available, there should be a recognisable latent/early symptomatic stage, there should be a suitable test/examination, the test should be acceptable, the natural history of the condition should be adequately understood, there should be an agreed policy on whom to treat, the economic costs of case finding and of providing care should be considered and case-finding should be a continuing process.^18^ We suggest these criteria are largely fulfilled by using the SDQ to screen for mental health problems in looked-after children. In particular, prevalence studies show high mental disorder rates within the looked-after population,^10^ indicating public health importance. Understandings of the natural history of child and adolescent mental disorders are increasing, with evidence that early symptoms can often be identified.^19^ Cost-effective, evidence-based programmes for particular groups, such as Multidimensional Treatment Foster Care^20^ and Attachment and Biobehavioral Catch-up for vulnerable infants,^21^ are available. In addition, the SDQ is a cheaper, shorter alternative to longer measures yet it has good sensitivity and specificity.^22^

More recently, it has been suggested that screening programmes should be evaluated in terms of the balance between their benefits (probability of an adverse health outcome without screening; degree to which screening identifies all those who suffer the adverse health outcome; health benefit of earlier versus later treatment) and harms (frequency and experience of those with false-positive tests or who are over-diagnosed; frequency and severity of harms of treatment).^16^ Weighing up this balance in the context of screening looked-after children requires acknowledgement of the potential stigma of a mental disorder diagnosis/label; in light of this, evidence-based interventions (e.g. enhanced foster care, enhanced sensitivity to foster infants, additional resources) may seem less likely to cause harm than treatments for screening-identified physical illnesses (e.g. surgery, radio- and chemotherapy for cancers). Again, screening for mental health problems in looked-after children appears justified.

### Do we have an effective screening programme in England?

Identification of those with problems is only the first step; the next is to address those problems. However, English local authorities have inadequate referral routes to CAMHS once the SDQ has identified children with possible mental disorders.^23^ The current programme of compulsory SDQ returns comes at a time when financial pressures mean many specialist teams offering support to looked-after children have been cut.^24^ The scheme incurs financial costs of its own and, despite the Department for Education's desire to improve routes to CAMHS, there is no mechanism to ensure abnormal SDQ results routinely lead to referral and treatment of identified individuals. The absence of such a mechanism is a policy-implementation deficit and we recommend renewed consideration of the programme, especially of referral pathways. Annual SDQ rates have remained remarkably consistent since the screening was launched, suggesting its introduction has not been associated with any change in the mental health of English looked-after children at a population level.

The expectation from the Department for Education is that these data are gathered annually,^9^^,^^25^ but high levels of missing data undermine this, with considerable variance in local authority completion rates in England. It is likely that these levels of missing returns relate both to understandings of the value of the data by some of those within local authorities involved in its collection, and the process of data collection itself. The latter involves encouraging completion by the child's carer, questionnaire collection, data entry and collation by local authority administrators, looked-after children specialist nurses or specialist looked-after children CAMHS practitioners. Although recent slight increases in rates suggest systems may be improving, we need to better understand why so many SDQ scores are missing.

There are ethical issues associated with continuing this policy in its current form if nothing is then done with these data to assess and support those CYP identified as having problems. Compulsory SDQ monitoring has enabled the scale of mental health problems to be identified among looked-after CYP and, as a public health intervention, there are benefits to regularly overseeing the mental health of a highly vulnerable group. Given the relative stability in these population-based data, there may be little benefit in continuing with the expense of data collection without first addressing the ethical and moral imperatives of the missing data and referral pathways to additional services for CYP who need support.

We argue that the current data collection is not achieving the screening programme aims and that some modifications of the existing system need to occur to improve the mental health of looked-after children.

At the time of writing (autumn 2018), baseline data on mental health are not routinely collected about CYP at entry to care. This could be construed as an oversight in the current system's design which could be remedied by incorporating it into the CYP's first medical. Investment in ten pilot sites that aim to improve mental health assessments for children entering the care system was announced in June 2018, as the Department for Education and Department of Health and Social Care accept that ‘looked-after children should undertake the SDQ as a starting point when they come into care, and then each year as part of compiling an accurate picture of their health needs’ (p. 6).^26^

### What could the data tell us beyond screening?

This mass data collection exercise might be useful for examining geographical variations or time trends, or as a performance indicator for local authorities.^4^^,^^23^^,^^25^ Data derived from the SDQ screening programme are only available for about 70–75% of children in any 1 year and approximately 40% of children move in and out of the care system each year,^27^ meaning that useful, representative, longitudinal analyses would be challenging – although not impossible if levels of missing data were reduced. This vast and annually increasing data set has great research potential: it is possible that lack of change at the population level masks real effects at an individual level and careful consideration of how individual analyses could be achieved should be part of any revision of the system.

### Suggestions for improvement of the current system

We suggest consideration of the further opportunities the annual SDQ data collection affords, both in terms of its analytic potential and as a screening programme. Currently the screening programme falls short, due to large amounts of missing data and no link to any ‘next steps’ for those children whose scores indicate likely disorder. As a data set, investment in better completion and more complex analyses may increase understandings of (likely reciprocal) associations between looked-after children's emotional/behavioural difficulties and both demographic and placement-related factors. Screening should not occur in isolation; investment in better systems would ensure SDQ scores for individual children are scrutinised, used in decision-making and – where they indicate likely psychiatric diagnosis – trigger clear referral pathways. These actions could result in improved placement and health outcomes for looked-after children, and this would be a worthwhile investment.

### Limitations

Our use of publicly available data and simple statistical analyses aimed to demonstrate time trends and examine representativeness. Some might argue that it is impossible to evaluate the impact of screening using the SDQ without conducting longitudinal analyses (e.g. comparing outcomes for those with/without an SDQ, or those coming into the system at earlier/later time points) or by examining proxy data on service referral rates, access and/or effectiveness as outcome indicators. We contend that examining population-level trends in SDQ scores offers insight into the impact of screening looked-after children, and that there are flaws inherent in any longitudinal analyses of incomplete data. Our simple analyses are thus an important first step in examining the SDQ screening programme.
